# Swelling of kappa carrageenan hydrogels in simulated body fluid for hypothetical vessel occlusion applications

**DOI:** 10.1177/08853282221110357

**Published:** 2022-07-01

**Authors:** Florian Wurm, Norbert Lerchster, Germar-Michael Pinggera, Tung Pham, Thomas Bechtold

**Affiliations:** 1 Research Institute for Textile Chemistry and Textile Physics, Faculty of Chemistry and Pharmacy, 151267 University of Innsbruck, Dornbirn, Austria; 2 Institute for Environment and Food Safety, Bregenz, Austria; 3 Department of Urology, RinggoldID:27280 Medical University of Innsbruck, Innsbruck, Austria

**Keywords:** Kappa carrageenan, hydrogel, simulated body fluid, swelling, contraception

## Abstract

The swelling ability of kappa-carrageenan (KC) hydrogels was investigated in simulated body fluid (SBF). The SBF mimics the ionic concentrations in the vasa deferentia of human males. The study clarifies if these hydrogels can be adjusted to occlude the vasa deferentia by swelling. For this purpose, swelling to twice the initial volume is desirable. In this study, hydrogels of different primary potassium concentrations, biopolymer concentrations and ethanol-exchanged gels, were immersed in SBF either directly or after drying (pre-dried). We measured the absolute and relative swelling degree, and the swelling rates of the gels. Extensive pre-drying leads to irreversible gel densification and absolute swelling magnitudes decrease. We found that immersion into the SBF also leads to potassium ion accumulation, and network restructuring in the hydrogels. This markedly increases the storage moduli of the gel networks. The ion content in the gel structures also directly affects the swelling speed, the fastest swelling occurred in ethanol-exchanged and pre-dried gels. We found that by pre-drying and potassium content adjustment, swelling of the hydrogels is sufficient to render KC hydrogels as a possible candidate for the occlusion of the vasa deferentia.

## Introduction

Swelling has been investigated and modelled for numerous hydrogel systems.^1–3^ Hydrogels are extensively investigated for medical applications in dental impressions,^4^ drug delivery,^5^ embolization,^6^and wound dressings as dialysis membranes,^7^ where function is partly dependent on swelling. κ-carrageenan (KC), dominantly used in food and beverage applications,^8^ can form physically-cross-linked hydrogels prone to swelling. Though the use of KC in medical applications is envisioned,^9^ few swelling investigations in physiological media were reported,^10,11^ and to the authors knowledge, none for purely physically cross-linked hydrogels.

KC is part of a family of alternating 1,3-α and 1,4-β-linked, unbranched, polysaccharides consisting of sulphated and non-sulphated galactose and 3,6-anhydrogalactose monomers. These moieties are statistically distributed. KC has an estimated content of 22% sulphate groups and 33% 3,6-anhydrogalactose units.^8,12^ The water-dispersible polymer forms double helices after solvation by heating and further aggregates to networks and gels, given sufficient biopolymer and ion concentration, e.g. potassium. Networks are found to form interlinking domains, which assemble to extended gel structures.

Swelling of KC gels, physically and chemically cross-linked, have been reported in numerous studies. These include KC networks in sucrose, acetone solutions,^3^ water and potassium chloride solutions^13–15^ and at different temperatures.^16^ Additionally, sorbitol and glycerine effects on KC gel swelling was determined.^17^ Glutaraldehyde cross-linked KC gels were swollen in sodium chloride, potassium chloride and calcium chloride solutions.^18^ Genipin was added to the hydrogels for optimum release behaviour in drug delivery applications^19^ and methacrylate moieties were added for photo-cross-linked KC gels in tissue engineering. The latter gels were swollen in Dulbecco’s phosphate-buffered saline or Dulbecco’s modified eagle medium.^10^ Glucan/carrageenan gels were investigated for wound healing applications, swelling upon wound exudate contact.^11^ These final two studies focused on physiological swelling investigations in specific simulated body fluids, both using Glutaraldehyde as chemical cross-linker. Other studies used KC and a secondary polymer, like polyvinyl alcohol or hyaluronic acid, composites for controlled release applications, partially based on hydrogel swelling.^20–22^ Micronutrient delivery was also targeted using KC gels alone.^23^ Other approaches even report ‘anti-swelling’ and use KC-Alginate hydrogels for pollutant removal.^24^

For a specific medical application, namely the occlusion of the vasa deferentia in males, the swelling behaviour of physically cross-linked KC hydrogels in vas deferens fluid is of interest. Ultimately, the vessel occlusion could act as a male contraceptive. Since permanent occlusions of the vasa deferentia induce secondary epididymal obstruction, a liquid permeable hydrogel is an approach to circumvent the development of obstructions.^25–27^ In view of the ionic composition of the vasa deferentia fluid, it may be wise to use a potassium selective polymers for physically cross-linked hydrogels.^28,29^ Two specific properties of the occlusive are of primary interest. Firstly, the mechanical stability, to withstand peristalsis and ejaculative forces, and secondly, pronounced swelling after introduction to ensure complete occlusion of the vasa deferentia. Swelling times are of minor interest, as ejaculation stresses can be postponed and peristaltic forces are low. Introduction of the gels is envisioned by a minimal invasive procedure, by distending the vasa deferentia, possibly using lubrication or other technical means. Such a procedure has also been applied in a related contraception approach.^30^

For the sake of simplicity, as well as for medical considerations, chemical crosslinking of the gel structures was avoided. It is assumed, that it is possible to swell pre-treated KC hydrogels to twice its initial volume. This estimate derives from urological considerations, based on micro-surgical expertise, to ensure occlusion, though tissue resistance might limit final swelling. Swelling can be adjusted by prior drying or ethanol exchange.^31^ If sufficient swelling and mechanical stability is achieved, gels could be a suitable candidate for vessel occlusion or embolization applications.

To specify and estimate the swelling of KC hydrogels in the vasa deferentia we immersed KC hydrogels in a specific simulated body fluid (SBF). For the sake of simplicity, we constrained the study to the ionic influences of the simulated vas deferens fluid. Proteinaceous or cellular influences were not included in the scope of the investigations. The SBF represents a particular shift in the ionic environment of the investigated gels. This should be a valid approach, as the ionic species in the fluid are expected to be the main driving factor for gel swelling. We did not include cytotoxicity, biocompatibility or histological studies, as the present investigation is to pitch the applicability of the hydrogels for the envisioned application.

## Theory

Swelling of hydrogels is known as a not purely diffusional phenomena. Shear moduli of the swelling systems influence the swelling response, and specific influences have theoretically been described for different shaped gels by Li and Tanaka.^2^ The solutions of the swelling equations are equivalent to the vibrational modi of solid spheres.^32^ These solutions include the adaption by a time dependent displacement. Swelling and drying of gels can be described by equation (1).^33^

(1) $$\frac{W_{\mathrm{eqs}}-W}{W_{eqs}}=\overset{\infty }{\underset{n=1}{\sum }}B_{n}\;exp\left(-\frac{t}{\tau_{n}}\right)$$

W_eqs_ is the equilibrium swelling mass, W the swelling mass at time t. B_n_ are constants for the specific elements of the solutions of the differential equations, of which each is described by a specific time constant τ_n_ for the exponential function exp. A common application of equation (1) is the parallel exponential kinetic (PEK) model, using *n* = 2. For large t, as in the case of primary influence of the first term (*n* = 1), higher order terms are usually dropped. Equation (1) simplifies further by using a logarithm to

(2) $$\ln\left(\frac{W_{eqs}-W}{W_{eqs}}\right)=\ln\left(B_{1}\right)-\frac{t}{\tau_{1}}$$

B_1_ is between 0 and 1 and depends on the ratio R = G/M of shear modulus G and longitudinal modulus M for a cylindrical gel by

(3) $$B_{1}=\;\frac{2\left(3-4R\right)}{\alpha_{1}^{2}-\left(4R-1\right)\left(3-4R\right)}$$

(4) $$R=\frac{1}{4}\left(1+\;\frac{\alpha_{1}\text{*}J_{0}\left(\alpha_{1}\right)}{J_{1}\left(\alpha_{1}\right)}\right)$$

α_1_ is the eigenvalue of the first solution of the differential equation, and is determined by the boundary conditions. J_0/1_ are the respective Bessel functions of the first kind. From the swelling relaxation constant of the gel the collective diffusion constant D_c_ can be derived.

(5) $$D_{c}=\;\frac{3\;a^{2}}{2\;\tau_{1}\alpha_{1}^{2}}$$

a is the linear gel size extension and is interpreted whether discs, cylinders or spheres are being described. The collective diffusion coefficient of cylinders is only two thirds of that for spherical gels at the boundary of the gels.^2^ Li and Tanaka found little difference between cylindrical and spherical gels. For the measurements undertaken, we have to limit the description of a drying process to be constrained to small drying deformations only.^34^

## Materials and methods

### Materials and simulated body fluid

κ-Carrageenan from Danisco S/A (Danisco SA, Copenhagen, Denmark now DuPont) was received in food grade. All other chemicals were purchased in analytical grade as follows: sodium chloride and calcium chloride (Carl Roth GmbH & Co. KG, Karlsruhe, Germany), potassium chloride (Riedel-de Haën, Seelze, Germany now Honeywell) and ethanol (EtOH) (Deuring GmbH & Co. KG, Hörbranz, Austria). Compounds were used as received.

SBF fluid was adapted close to ionic concentrations of calcium (Ca^2+^), potassium (K^+^) and sodium (Na^+^) levels present in the vasa deferentia of human males, 1.5 mM, 100 mM (instead of 111 mM) and 30 mM respectively.^28,29^

### Molecular characteristics of kappa-carrageenan and swollen hydrogel ion content

Inductively Coupled Plasma Optical Emission Spectroscopy (ICP-OES) was used to determine ion contents of KC and the final swollen hydrogels, following DIN EN ISO 11885 standards. Wet-ashed sample solutions, following the lead determination procedure,^35^ were analysed in an argon-run Varian MPX (Palo Alto, CA) by spraying into a 0.5 L/min, 0.3 mPa pre-pressured, gas flow. High frequency generator power was set to 1.15 kW. Calibration was performed via a 12-point routine. Three independent measurements were averaged to determine concentrations.

Viscosity measurements were used to determine molar mass estimates.^36^ In short, KC was hydrated and dissolved by short heating to 70°C in 0.1 M sodium chloride solution. After heating a second time to 70°C on the following day, samples were left to cool and then analysed under averaging shear rates between 150-300 Hz.^37^ Using a = 0.86 and K = 8.84 × 10^−5^ dl g^-1 38^ number average molar mass estimates were derived using the Mark-Houwink relation $\left[\eta \right]=KM_{w}^{a}$.

Sulphate content of the KC was determined in accordance with DIN ISO 22743 in a non-fluent system.^39^ After wet-ashing the biopolymer in 1 M hydrochloric acid for 8 h at 100°C, diluting and neutralising it, the sample was mixed with a methylthymol blue (MTB) – barium chloride solution. Finally, photometric extinction of 445 nm light was used to determine the non-complexed MTB and thereby the sulphate content.

Determination of the KC monomeric composition was carried out via proton nuclear magnetic resonance spectroscopy (1H NMR). A 600 MHz Bruker Avance II+ (Billerica, MA) with a Prodigy TCI Probe was used. 32 scans at 65°C with an interscan delay of 5 s were conducted. 4,4-dimethyl-4-silapentane-1-sulfonic acid (DSS) was added as internal reference (δ = 0 ppm). 0.5% by mass in deuterium oxide (D_2_O) was ultrasonified and stirred for 120 min at 40°C to decrease sample’s viscosity before analysis. Peak areas of the signal for the anomeric hydrogen on the anhydro galactose residues were determined and compared. For μ- and ν-carrageenan fractions, the anomeric hydrogen of the sulphated galactose was used.^40^ Errors were derived from the uncertainty in the peak fitting routine and carefully estimated to 1%.

### Hydrogel production, drying and swelling procedure

1–3% by mass biopolymer solutions were prepared by addition of the dry KC powder to deionized water. Calcium chloride, potassium chloride and sodium chloride were added to obtain 1.5 mM calcium and 30 mM sodium gels. Default potassium level was 100 mM, but gels of 50 mM and 300 mM were also used. Mixtures were heated to 80°C, 300 mM samples even to 95°C, for 25 min, to ensure complete dissolution of the biopolymers. Hot solutions of (10 ± 1) g were poured into two different types of glassware to produce gels. Gels for rheometric analysis were poured into 60 mm diameter beaker and were of flat disk shape (V = 0.118 * r^3^ * π). These are named disk samples below. The other gels were derived by pouring solutions in 35 mm diameter beaker. For these gels’, heights were about two thirds of their radii (V = 2/3 * r^3^ * π). For distinction purposes these are named cylinder samples below.

Gels were either used directly for swelling measurements, or they were pre-dried in an oven at 50°C for the time required to dry initial gels to about (50 ± 8)% of their mass (see Table 1). Ethanol samples were immersed three times in pure EtOH, with increasing immersion times, however at least 1 h. Each gel was immersed in 200 mL SBF at room temperature, and left between mass determinations without stirring to minimize mechanical damage of the gel matrix. SBF was changed upon every mass determination. Gels were immersed in SBF until equilibrium swelling was perceived.

**Table 1.** Hydrogel formulations, given by kappa-carrageenan and potassium concentration alterations, of the investigated gel swelling as their pre-treatment before immersion in 200 mL simulated body fluid (1.5 mM Ca^2+^, 100 mM K^+^, 30 Na^+^).

Sample set	Biopolymer (% by mass)	Potassium (mM)	Pre-treatment
Primary	1	50, 100 and 300	None
Dried	1	50, 100 and 300	Oven drying (50°C) to ∼50% by mass
Concentrated	1, 2 and 3	100	Oven drying (50°C) to ∼50% by mass
Ethanol	1, 2 and 3	100	3-times ethanol immersion, followed by oven drying (50°C) to ∼50% by mass

### Rheometric characterization

Analysis was performed on an MCR302 (Anton Paar GmbH, Graz, Austria) using a serrated parallel plate system. Gels underwent an initial reference phase (120 s, 10 Hz, 0.3% amplitude), a frequency sweep (100–0.01 Hz, 0.3%), another reference phase, an amplitude sweep (0–20% in 1% steps, then 10% steps up to 500%, 10 Hz) and a final reference interval. Normal force was below 2 N. Measurements were at room temperature, which was well below gelation temperature. The serration of the plates prevents wall slippage, though it introduces non-homogenous shear contributions on the gels.

## Results and discussion

### Molecular properties of kappa-carrageenan

Ionic content, molar mass, sulphate content and KC composition estimates are reported in Table 2.

**Table 2.** Specifications of contained ion concentration for 1% solutions (±10% error), molar mass estimate, sulphate content and carrageenan-type composition (±1% error) of the used kappa-carrageenan.

	Ca^2+^ (mM)	K^+^ (mM)	Mg^2+^ (mM)	Na^+^ (mM)	M_w_ (kg/mol)	SO_4−_content (%)
KC (Grindsted CW950)	1.20	20.74	1.22	2.53	635 ± 55	23.9 ± 1.2
		κ (%)	μ (%)	ι (%)		
KC (Grindsted CW950)		87	3	10		

KC: kappa-carrageenan.

The potassium salt of KC present coincides roughly with estimates for an average sulphate content of KC.^12^ As found in ^1^H-NMR a 10^th^ part of ι-carrageenan is present in the KC used, with a minor share of μ-carrageenan. Constituent carrageenans were estimated integrating Lorentzian shaped peaks after deconvolution of the NMR signal using peak centre estimates.^40^

### Drying of hydrogels

**Figure 1.**
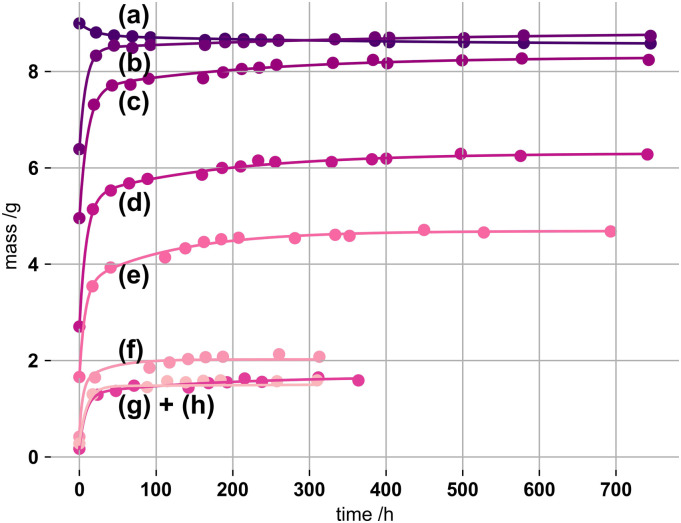
Swelling masses of kappa-carrageenan gels (1% by mass, initially 100 mM K^+^) immersed in simulated body fluid, oven dried (50°C) for (a) 0 h, (b) 1.1 h, (c) 3.6 h, (d) 5.1 h, (e) 6.75 h, (f) 9.75 h, (g) 12.75 h and (h) 22.6 h. PEK-exponentials were fitted using equation (1) constraining *n* to 2.

An initial experiment was undertaken to establish a method for partial gel drying, and assess the limit of reversible swelling. Cylinder samples were oven dried, at 50°C with increasing duration, before immersing in SBF. Figure 1 shows the respective swelling of the pre-dried gels.

Swelling of the network terminates once further imbibition of fluid is zero. As seen from Figure 1 gels of longer drying periods suffer from low recoverability. During the drying process the network structure must have undergone irreversible changes and partial densification.^33^ After swelling, prior gel masses are not reached. Besides, equilibrium swelling is more rapid for lighter and smaller gels than for heavier and larger structures. This is diffusion dependent as volumetrically bigger gels need more time for equilibration.

**Figure 2.**
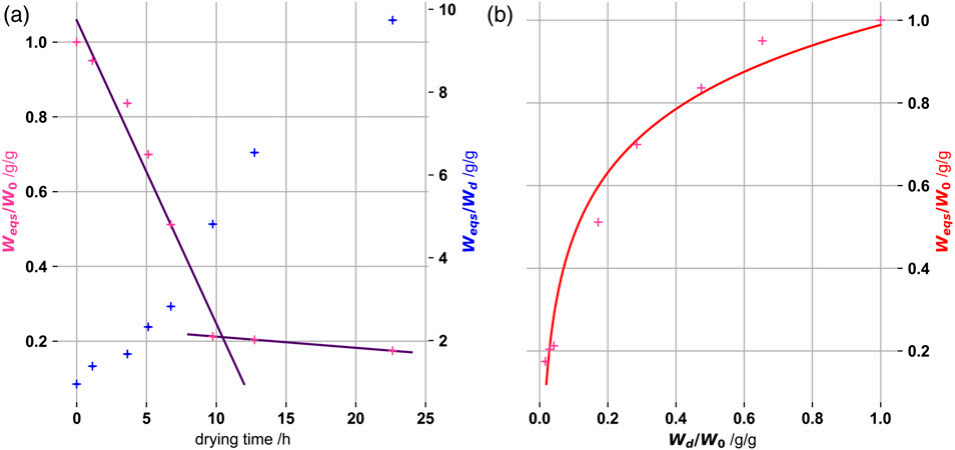
(a) Ratios of W_eqs_ to W_0_ (pink - left axis) and W_eqs_ and W_d_ (blue - right axis) with respect to drying times (b) Ratios of W_eqs_ to W_0_ with respect to the ratio of W_d_ to W_0_. Provided functions were fitted to guide the eyes. Kappa-carrageenan gels were 1% by mass and 100 mm K^+^.

Ratios of equilibrium swelling masses (W_eqs_) to pre-drying masses (W_0_) (Figure 2(a)), with respect to drying time, and dried to pre-dried masses (W_d_/W_0_) (Figure 2(b)), are shown in Figure 2. All pre-drying masses in the ratios were corrected for the decrease due to ionic accumulation in the gel, as present in the reference gel.

For drying times of more than 5 h substantial degenerative changes of the network structures appear. Upon longer drying gel equilibrium swelling (W_eqs_) are less than about 2/3 of pre-drying values (W_0_). For less than 5 h, gel equilibrium swelling (W_eqs_) reduction is below 1/3 of pre-drying (W_0_) values and we assume little irreversible changes, i.e. due to capillary collapse, of the network’s structures.

Drying linearity is expected for sample drying,^34,41^ though the investigated gels are non-spherical. We assume that the cylinder samples’ drying behaves similar to spherical hydrogels. However, this is contrary to the description using equation (1).^2,33^

It seems the drying curve up to 10 h shows convex behaviour. This indicates that the drying flux increases with drying time.^33^ Bertrand et al. also reported this, assuming that linearity is just an approximated behaviour of the real water transport dynamics in drying gels. Moreover, porosity on the surface of the gel changes, altering the drying flux of the gel due to an increasing amount of escaping solvent molecules.^34^ During drying, the polymeric chains relax from the elastic stress and help to expel water from the gel.

Wu et al.^33^ theorised that physically bound gels restructure upon distance decreases between polymer molecules due to solvent escape. This increases cross linking density and reduces interchain spaces. Bertrand et al.^34^ reported that after a certain drying time, masses reach a plateau for which further mass loss is close to zero. The second linear fit (purple line) in Figure 2(a) has a slight negative slope, indicating that final drying was not reached after 12.75 h. Due to the lack of values above 22.6 h and the low number of data points a specific slope for the linear fit is not given. We also could not deduce the point of drying cessation. Eventually, drying levels out for longer drying times.

Even though the equilibrium swelling masses (W_eqs_) are smaller for longer pre-drying times (see Figure 2(a)), these degraded gel structures have the highest mass swelling ratio (W_eqs_/W_d_). We assume this ratio will plateau out, as the drying, and the collapse of the gels also plateaus.

It is reasonable to assume that the interchain spacing changes will result in degenerative changes in the gels (Figure 2(b)), reducing equilibrium swelling mass (W_eqs_) of gels below the pre-drying values (W_0_). We expect most collapsing and degenerative changes in the gel to appear, after half of the pre-drying mass (W_0_) has evaporated off. We therefore limited the drying of the gels in the following measurements to 50%, assuming that most of the degenerative changes have not yet appeared. It can be inferred, that 50% drying has little impact on the gel structures, and that swelling kinetics are due to differences in gel compositions. The swelling amplitude increases due to drying, enhances the accuracy of the measurements as their model descriptions.

**Figure 3.**
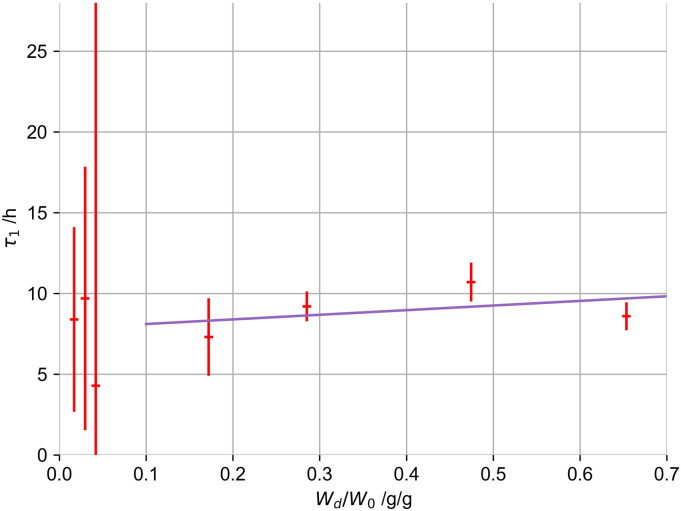
Changes in the first swelling time constant of gels (1% by mass, 100 mM K^+^), dependent on the degree of drying (W_d_/W_0_).

The changes in τ_1_ (obtained from Figure 1), the first and dominant term of the PEK model, of the gels with respect to drying times can be compared, as shown for selected drying degrees (W_d_/W_0_) in Figure 3. These changes are a cumulative effect of the degree of drying and the structural changes induced, which hinder diffusion processes.

A linear relation was used to correct the specific swelling timescales of all measured gels to a drying state of exactly 50%. We know that this relation might only partly be true for gels with higher primary biopolymer concentration or different potassium concentrations. We nevertheless estimate the drying influence on gel structures as primary influence that has to be accounted for. We accept the minor errors introduced by this correction for non-1% and non-100 mM potassium gels.

### Swelling of hydrogels

We immersed one set of samples (50 mM, 100 mM and 300 mM potassium), the primary samples, without pre-drying into SBF. We further prepared dried samples from different potassium ion concentrations, different concentrations of biopolymer (1%, 2% and 3%), and we exchanged one set of hydrogels (1%, 2% and 3%) with ethanol. Though ethanol is a non-solvent to carrageenans, by pre-treating gel structures with ethanol usually the structural changes during xerogel production are reduced, and higher structural integrity is preserved.^42,43^ The post solvent-exchange masses (W_0_) of EtOH gels were ∼30% of initial gel masses. Similarly, 30–40% volume (for simplicity we assume unity density) were recorded in a related study using KC.^31^ Gel volume decreases to 20–40% were also reported for Na-alginate beads above 20–30% v/v ethanol mixtures, dependent on ionic strength.^44^

**Figure 4.**
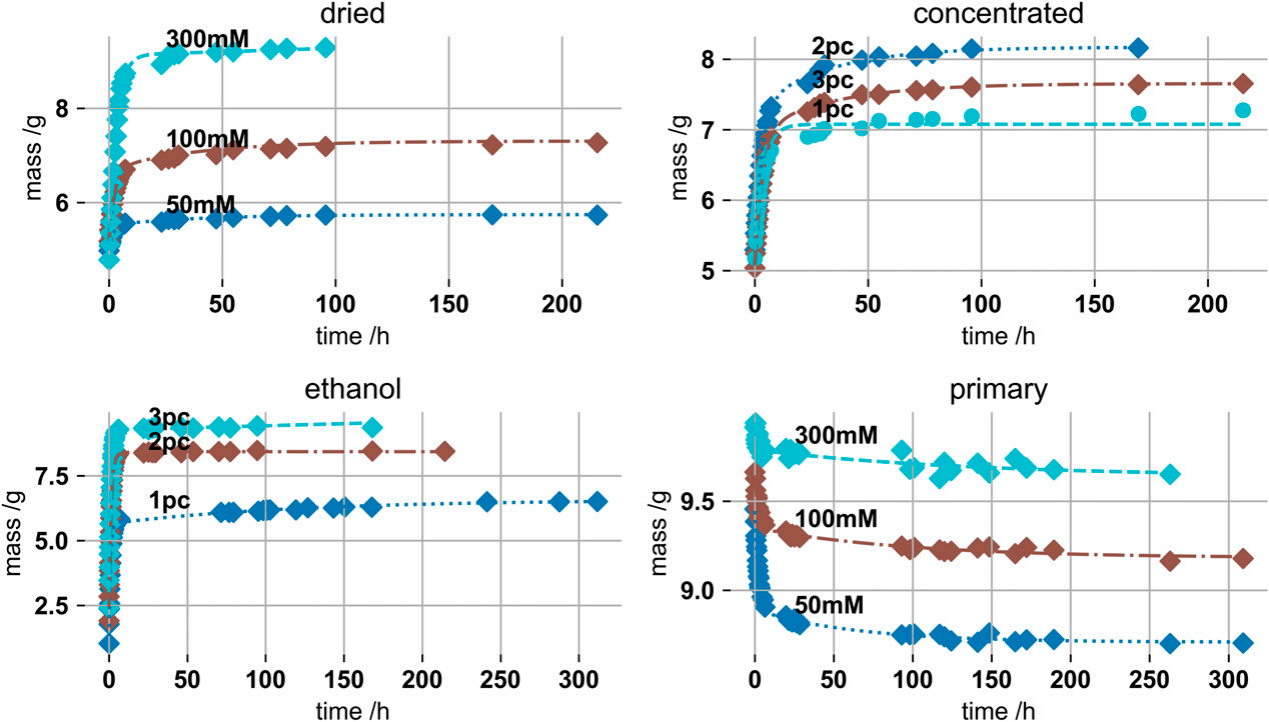
Swelling masses of (top-left) 1% by mass 50, 100 and 300 mM potassium dried gels, (top-right) 1–3% by mass, gels of 100 mm potassium, (bottom-left) 1–3% by mass gels, 100 mM potassium after pre-ethanol exchange and (bottom-right) 1% by mass, 50, 100 and 300 mM potassium primary gels.

Figure 4 shows swelling mass data of these four systems as a function of swelling time. PEK exponential functions were fitted. Drying was driven to about (50 ± 8) % of the initial gel mass. This is one point of criticism of the study. We assumed the reversibility and drying influence of gels of 2 or 3% by mass or 50 and 300 mM potassium to behave similar to 1% by mass and 100 mM KC gels. The gels, apart from the non-dried ones, re-swell upon immersion in the SBF. The primary samples densified due to potassium ion accumulation.^4,44^ Ethanol samples show the highest absolute swelling changes, from 1.03 to 6.5 g for a 1% and 2.4–9.4 g for a 3% gel respectively, which relate to swelling of more than 300% by mass.

Drying and ethanol exchange enable the observation of substantial swelling changes. In the final application, hydrogel pre-drying and ethanol exchange can be performed prior to vas deferens insertion. Besides after drying, most of the ethanol is evaporated off. After minimal invasive insertion, extensive swelling inside the vasa deferentia possibly enables thorough occlusion.

Several factors drive the swelling changes. The electrochemical potential due to unsaturated anionic sulphate groups drives the diffusion of potassium ions into the hydrogels. This eventually equilibrates with osmotic pressure, driving potassium ions out and solvent molecules into the gel. Apart from that, gel structures and polymers inherently resist to stretching, as this requires energy, further slowing diffusion of solvent molecules into the gel.

**Figure 5.**
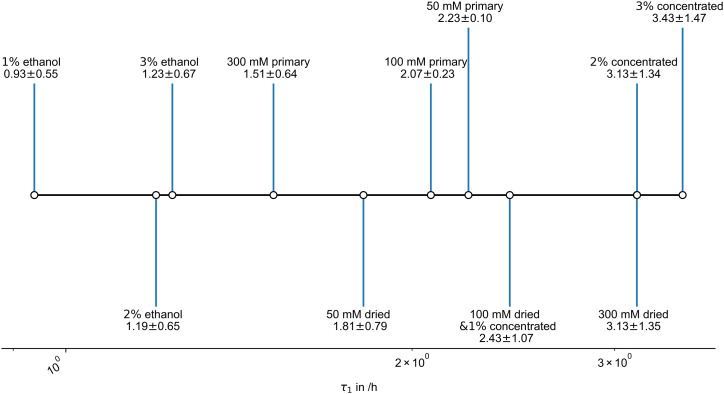
Respective first, and primary, parallel exponential kinetic swelling time constants of the different gels in simulated body fluid.

Specific swelling time constants τ_1_ for the first element of the PEK model can, after correction, directly be obtained from the swelling fits (Figure 5).

The first timescales of the swelling changes of the primary gels decrease with an increase in potassium concentration, as the ions drive hydration and osmosis. Though the overall extent of swelling changes are low for higher potassium gels, these happen faster.

Dried gels show increasing swelling timescales with increased potassium ion content. This is probably driven by the restraint of gel expansion by the ions. Higher biopolymer concentrations possibly also restrain the swelling time scales, however 100 mM dried and 2% and 3% by mass concentrated gels cannot be separated by values.

The different ethanol-exchanged carrageenan gels show minor changes in swelling speeds, presumably with biopolymer concentration. Overall swelling is multiple times faster than for the other swelling gels. Ethanol pre-exchanged gel systems are highly reversible-swellable. If we compare W_eqs_ masses with W_0_ for the gels, we find that they are 76%, 89% and 92% for a 1%, 2% and 3% gel respectively. This accords with densification due to accumulation of potassium ions and is higher than for gels without ethanol exchange. Accordingly, it is concluded that structural changes, including pore collapse, during drying are suppressed for ethanol gels. This has been reported for various gels and systems.^43^

The obtained timescales are difficult to compare with given literature values.^13,14,16^ 19 × 4.5 mm gel discs swollen in pure water and KCl solutions exhibited swelling timescales of 12–88 min^13,14^ While KC gels in water vapour resulted in 86–298 min^16^ These studies used a minor share of gel volumes, complete gel drying which generates structural restrictions, and different ionic concentrations. For the final application, the absolute swelling time of a few hours is sufficiently fast. After gel insertion into the vasa deferentia, sexual abstinence and non-ejaculation prescription enables sufficient swelling before increase mechanical forces exert on the hydrogels. Intra-vasal peristalsis is assumed insufficient for gel expelling.

**Figure 6.**
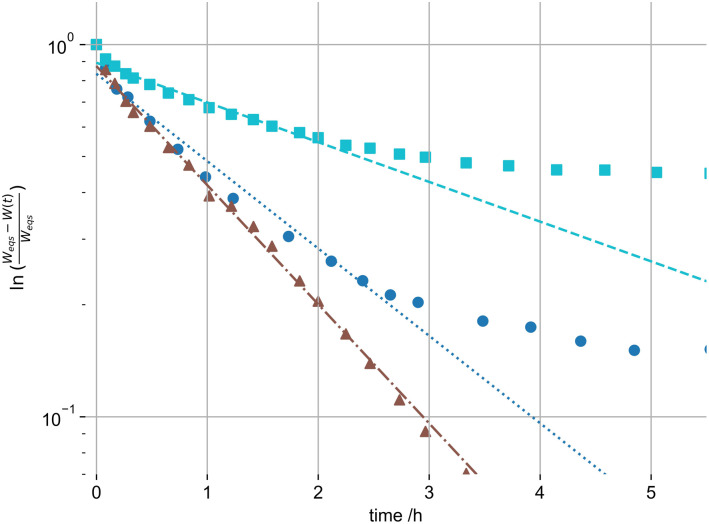
Representative linear fit of swelling of hydrogels of the logarithmic swelling change in 1% (dotted - circle), 2% (dotted-dashed - triangle) and 3% (dashed - square) by mass ethanol swelling gels.

**Figure 7.**
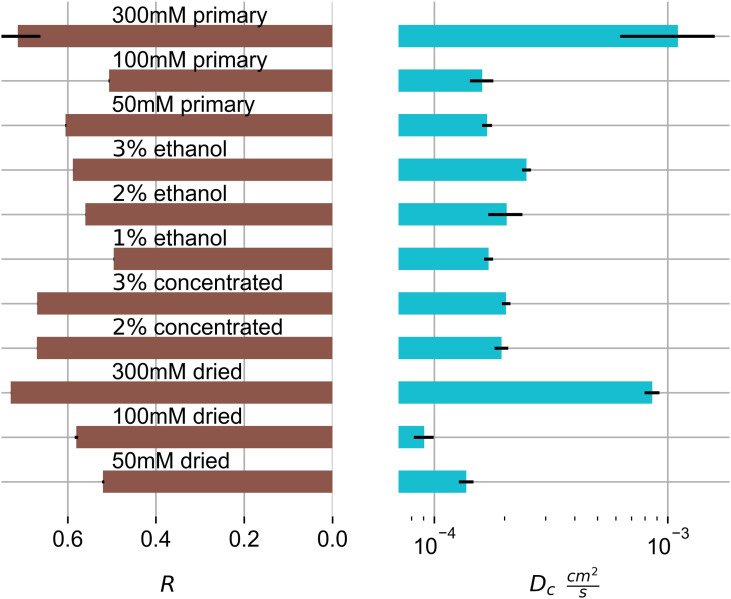
Horizontal bar graph for the calculated R and the collective diffusion coefficient D_c_ of the swelling hydrogels. Black bars represent σ-errors.

Using equation (2) we fitted gel swelling as a function of time to derive the B_1_, R and D_c_. For ethanol samples this is shown in Figure 6 and values are shown in Figure 7. The linear approximation in Figure 6 is difficult where the magnitude of the second term of the PEK model reaches 40% of the first term, as the assumption of neglecting all higher terms is questionable. This was the case in only 3 swelling systems out of the 12 investigated. Accordingly, we have screened and selected the data points for linear fitting for each system. We have neglected higher terms for comparison of the primary swelling influence, as due to the difficulty of their interpretation. This primary term is the most weighty, and represents the fast initial swelling. It is also dominant in 9 out of 12 gels, and becomes discussable for 3 of the 12. Hereby it is the additional slower swelling processes that will alter the derived R and D_c_. This alteration is expected small however.

From the R data we can deduce that the KC gels show shear moduli which are between 50 to 75% of their respective longitudinal moduli. R = G/M can be rewritten as R = G/(K+4 G/3), using the bulk modulus K. Depending on the gel R can range from 0 to ¾,^2^ which is the case for primary 300 mM gels. This indicates that the swelling behaviour of these gels is extensively shear driven. It also has the highest collective diffusion coefficient.

300 mM potassium gels have the highest collective diffusion coefficient, even higher than the diffusion coefficients of the 100 mM potassium ethanol samples. Unluckily, 300 mM potassium ethanol gels were not included in the analysis. Ethanol samples seem to show a minor dependency on the primary biopolymer concentration, at least the 3% by mass ethanol gel has a higher D_c_ value than the corresponding 1% by mass ethanol gel. Relating to all gels, there seems to be a lower difference in diffusion coefficient for altered biopolymer concentrations. Changes in the diffusion constants seem to be primarily driven by potassium concentration alternations.

Sason et al. reported that immersion of KC gels in ethanol decreased the potassium concentration in the gels, even to values below the critical gelation regime.^31^ This indicates that post-ethanol immersion, the gel potassium concentrations are depleted. Potassium accumulation in KC gels is expected, once ethanol gels, most of the ethanol being evaporated off, are immersed in SBF. If the swelling speed is enhanced by electrostatic attraction of the SBF’s potassium ions or the carrageenan chains cannot be concluded. It is likely though, that by diffusion of the potassium ions into the gels, they drag along a hydration shell and thereby increase swelling speeds. Comparing ethanol gels with other dried gels, we also assume diffusion to be faster due to less restrictive gel structures after drying.

Several studies reported collective diffusion coefficients found in completely dried KC gels. These included swelling in pure water,^14^ in potassium chloride solution^13^ as in water vapour.^16^ The found D_c_ values in the range of 10^−5^ cm^2^/s for pure water and potassium chloride solutions and 10^−6^–10^−7^ cm^2^/s in water vapour. The differences between these studies were due to the differences in solvent and vapour penetration in gels.^16^ The differences to our studies probably derive from the comparably low calcium, potassium and sodium concentrations (2.5%, 5% and 1% respectively) used in these studies, as the complete drying of the gels. The latter introduces swelling hindrance by structural gel changes. The collective diffusion coefficient also matches with the self diffusion constants reported for nano particles.^45^ The values found in this study, from 10^−5^ to 10^−3^ cm^2^/s, for the different gels in SBF therefore seem reasonable. We want to note, that the diffusion coefficient is an average of the position dependent diffusion coefficient, and should therefore only be regarded as an estimate. Besides, it is only used as a description of swelling of gels based on prior drying and exposure to additional potassium ions.

Returning to the contraceptive, from the data above, the swelling magnitude can be tuned to a factor of 2, possibly more with additional pre-drying. Specifically, the EtOH exchange also boosts the swelling magnitude. It is therefore assumed, neglecting tissue pressures and resistance, that lumen filling hydrogel structures can be produced. The latter is especially important with consideration of the lumen folds, which could enable semen leakage.

### Viscoelasticity comparison

Disk-shaped gels were swollen in SBF. Post equilibrium swelling, gels were investigated by rheometric analysis. The storage moduli of the gels (Table 3) were derived from averaging the initial 2 min 10 Hz data. We estimated the error carefully to 5%, which is an estimate derived from experience.

**Table 3.** Storage moduli of the different equilibria swollen kappa-carrageenan gels for 0.3% shear at 10 Hz. Non-swollen values are without swelling simulated body fluid immersion.

1% biopolymer concentration			
G (±5%) (Pa)	50 mM potassium	100 mM potassium	300 mM potassium
Non-swollen	3980	6770	15,130
Primary	6400	9530	13,900
Dried	11,370	12,360	13,860
100 mM potassium concentration			
G (±5%) (Pa)	1%	2%	3%
Non-swollen	6770	20,370	39,000
Concentrated	12,360	24,360	51,000
Ethanol	10,710	45,700	55,260

Primary samples of 50 and 100 mM undergo strengthening due to immersion in SBF,^44^ whereas 300 mM gels seem to be mostly unaffected. Comparing primary samples and samples pre-dried before immersion, we find potassium ionic samples of 50 and 100 mM to show increased storage moduli due to the drying process. The same is true for gels of different biopolymer concentration. This is partly expected, as drying induces restructuring of the gels. 300 mM samples by comparison show little difference in terms of stability. Probably, the higher amount of potassium ions hinders restructuring of the gels during drying. No obvious difference in the gels in amplitude sweeps can be observed. Concentrated and ethanol treated samples showed no significant differences of storage moduli.

**Figure 8.**
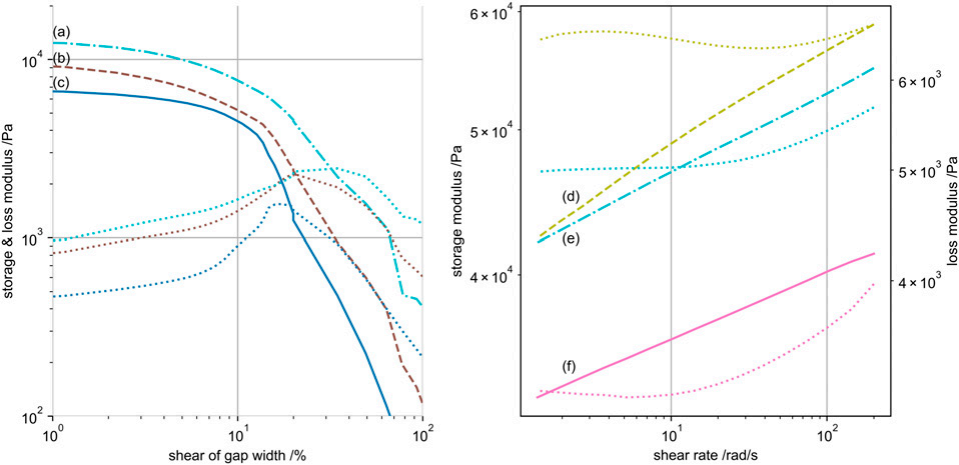
(a - left) Shear response of differently treated 100 mM potassium 1% by mass kappa-carrageenan gels. (a) Dried (dash-dotted), (b) primary (dashed) and (c) non-swollen (solid) samples. Respective loss moduli are of same colour dotted lines 8 (b - right) Shear rate response of 3% by mass samples after swelling for (d) ethanol-exchanges (dashed), (e) dried (dash-dotted) and (f) non-swollen hydrogels. Same colour dotted lines represent the respective loss moduli in both figures, for which Figure 8(b) has a secondary axis on the right.

Changes in crosslinking and restructuring of the networks is also changing the gels’ behaviour during shear.^31^ A qualitative example for the shear response changes of 100 mM KC gels is given in Figure 8(a) and for the shear rate of 3% by mass samples in Figure 8(b).

We refer to a collapse of the elastic gel, once the storage and loss modulus equal for increasing shear magnitude.^46^ This critical shear at storage-loss modulus (G’-G’’) equality is used as comparable quantity. First, we focus on the different 1% by mass KC gels. For 100 mM nearly no difference in critical shear (∼20%) is present for non-swollen, primary or ethanol gels. This might indicate little structural changes in terms of biopolymer aggregation. However, this critical shear increases (∼30%) once the gels undergo an aqueous drying step, probably inducing structure changes by capillary forces. For 300 mM gels, the differences in critical shear (all ∼30%) vanish, even for dried gels. Contrary, dried 50 mM gels show high critical shears (∼70%), while primary gels collapse very fast (∼15%). This could be interpreted as potassium induced hardening of small structured hydrogels, compared to drying induced structure opening. This accords with the similarity of critical shears of 300 mM gels, where due to the presence of potassium, the drying force do not alter the gels sub-structures. Above mentioned gels show a super-logarithmic increase in storage modulus with shear rate. Only the non-swollen and dried gels of 300 mM respond below a logarithmic increase.

Second, we compare gels of increasing biopolymer concentration. Though overall gel elasticity increases with amount of biopolymer, the critical shear decreases by a factor of 2 for non-swollen and dried 3% by mass gels, and even by a factor of 4 in 3% by mass ethanol gels. These higher concentrated gels behave similar to 1% gels with increasing shear rate. Only the 3% by mass ethanol gels responds over-proportional with increasing shear.

With respect to the final application, the required mechanical rigidity is difficult to assess. No study about peristaltic forces inside the vas deferens was found. Weak regular contractions of the vas deferens are reported, enabling injections during ejaculations in dogs applying 4 kPa pressure.^47^ Comparing with the esophagus, peristaltic forces of 16 kPa could arise. However, the soft contractions are also perceived in surgery, and expected substantially weaker than esophagus. Accordingly, the gels used in this study, especially the 2% and 3% by mass KC gels, should be sufficient in the use case. However, this is a hasty conclusion, neglecting chemical degradation and desired application duration.

### Gel equilibrium ion content

The determination of the ion concentration, limited to sodium and potassium ions, inside the hydrogels was undertaken after final swollen gel wet ashing. The respective ion concentrations found in the different gels are listed in Table 4.

**Table 4.** Ion content (±10% error) of wet-ashed swollen hydrogels.

	Reference	1% ethanol	2% ethanol	2% dried	3% dried	50 mM dried	50 mM prim	100 mM prim
Sodium (mM)	0.56	22.55	30.19	29.13	33.68	20.16	30.53	31.14
Potassium (mM)	0.08	87.37	125.09	121.39	150.01	79.13	115.09	117.60

Possibly, drying depresses the ion content in the final hydrogels. However, since no comparison with primary ethanol gels nor 100 mM dried samples is possible, this indication solely bases on the 50 mM primary to 50 mM dried sample, which is insufficient for a substantiated guess. Nevertheless drying induces irreversible densification of the network. Besides, gels of equivalent biopolymer concentration accumulate equivalent amounts of ions over the swelling treatment, given drying or non-drying procedures during production.

## Conclusions

Several KC gels were swollen in a simulated body fluid that mimics the ionic concentration of the fluid in the vasa deferentia in males. The swelling of the gels can be described by the model of Li and Tanaka.^2^ The relation of degenerative changes in the gel due to drying times was determined. Using pre-drying, the extent of swelling can be adjusted. Dependent on primary potassium ion concentration, gels undergo densification and strengthening upon immersion in the SBF. This results in higher storage moduli and higher strains at break. Hydrogels that have been pre-exchanged with ethanol exhibit faster swelling than pre-dried gels of equivalent concentrations. The concentration of potassium ions has a higher influence on swelling speeds than the biopolymer concentration.

There are several notes and limitations to the studies conducted. Long times between measurements do not conflict with the validity of the conclusion drawn. It would be of interest to prepare higher than 300 mM potassium gels, to see if inverse swelling appears. However, the production methods used, constrained preparing homogenous gels of higher potassium concentrations. The drying of the different gels should be unified, as that changes the possible equilibrium swelling ratio. If given, one could also compare absolute swelling differences of the gels.

The influence of the vasa deferens tensioning and peristalsis, and hereby the pressure counteracting on the swelling, have been neglected in this study. We assume hydrogel filling of the lumen, as the folds of the pseudostratified epithelium, possibly its flattening. However, meaningful estimation of this topic necessitates several experiments, including an adequate model if possible with peristaltic, stress-strain characterisations and eventually animal vessels. Further more biocompatibility, cytotoxicity and follow-up studies have to be undertaken. Accordingly, we see all this as parts of a follow-up investigation.

From the observed swelling of the gels, we can conclude that by potassium changes, drying and pre-treatment with ethanol, the absolute swelling degree, as the swelling speed, can be adjusted to desired values. Assuming unity density, doubling the hydrogel volume is possible using pre-treated KC gels. Pre-dried gels will consequently expand in size in the vasa deferentia and ensure complete occlusion, besides showing high mechanical stability. From swelling characteristics we therefore consider these gels are a possible candidate for vas deferens occlusion. However, substantial tissue forces and swelling resistance has to be considered, and investigated in future studies. This can however be partly counteracted by increases of the KC concentration in the hydrogels.
